# Arthroscopic excision of separated ossicles of the lateral malleolus

**DOI:** 10.1007/s00776-013-0412-3

**Published:** 2013-06-01

**Authors:** Satoshi Monden, Atsushi Hasegawa, Naohiro Hio, Masanori Taki, Hideo Noguchi

**Affiliations:** 1 Kiryu Orthopedic Surgery Hospital, 284-1 Hirosawacho-Ainoshima, Kiryu, Gunma 376-0014 Japan; 2 Higashimaebashi Orthopaedic Clinic Center of Foot & Ankle Surgery, 1317-3 Nishi-Ohmuro, Maebashi, Gunma 379-2104 Japan; 3 Ishii Orthopaedic & Rehabilitation Clinic, 1089-1 Shimo-Oshi, Gyoda, Saitama 361-0037 Japan

## Abstract

**Background:**

We have conducted a retrospective review of 19 patients for whom 20 separated ossicles of the lateral malleolus were excised arthroscopically. We examined the operating methods, findings, and overall results.

**Methods:**

The patients’ indications for this procedure were as follows. The main complaints were pain alone; ossicle sizes were small and ankle instability was minimal. There were 12 ankles of 12 males and eight ankles of seven females. The patients’ average age was 17.6 years. A 2.7-mm, 30° arthroscope was inserted into the ankle joint through the anterolateral portal. Instruments were inserted through the accessory anterolateral portal, and ossicles were removed piece by piece. Talar tilt angles and anterior displacements were examined and compared before and after surgery by use of stress radiographs. Japanese Society for Surgery of the Foot (JSSF) ankle/hindfoot scales were assessed pre and postoperatively.

**Results:**

All patients recovered their original levels of activity. The mean talar tilt angle changed from 6.1° ± 2.4° preoperatively to 6.0° ± 1.8° postoperatively (*p* = 0.93), and the mean anterior displacement changed from 5.9 ± 1.7 mm preoperatively to 6.1 ± 2.0 mm postoperatively (*p* = 0.42). Average JSSF ankle/hindfoot scale improved from 77.6 ± 2.6 points preoperatively to 97.2 ± 5.2 points postoperatively (*p* < 0.01).

**Conclusions:**

Arthroscopic excision of separated ossicles of the lateral malleolus achieved good results with minimum incisions, and relatively early resumption of daily and sports activity was possible. However, when the ossicles were embedded within the fibers of the anterior talofibular ligament, it was impossible to avoid cutting of ligament fibers. To reduce the possibility of ligament dysfunction, we believe postoperative treatment should conform to the accepted method for treatment of acute ankle sprains.

## Introduction

Among patients who complain of pain at the tip of the lateral malleolus after ankle sprains or sports activity, separated ossicles (“os subfibulare”) are often noted. These ossicles are found in 1 % of the human population [1], and are a result either of an unfused accessory ossification center [2] or an avulsion fracture of the anterior talofibular ligament [3, 4]. The presence alone of separated ossicles does not always cause symptoms. However, they can become symptomatic during trauma or overuse because of exercise, and require treatment [4]. Lateral ankle pain is believed to be caused by traction stress of the ossicle from the attached ligament [5] or by surrounding synovitis and hypertrophic soft-tissue impingement [6]. In general, nonoperative treatment should be chosen first (two to four weeks of rest with restricted weightbearing on crutches or immobilization) [2, 7]. However, surgical treatment can be required for cases which resist nonoperative treatment and for which symptoms recur. In operative treatment, excision of the ossicle (and repair or reconstruction of the lateral ligament of the ankle) and fusion of the ossicle in open treatment are performed, and good results are usually obtained [2, 3, 5, 7]. We selected fusion of the ossicle in cases where the size of the ossicle was relatively large and the patient was young (before completion of epiphyseal closures) and achieved high success of bone union. We performed excision of the ossicle (and repair or reconstruction of the lateral ligament of the ankle) in other cases, especially when the patient desired to return as early as possible to sports activity. Among patients requiring surgical treatment, there could be indications for arthroscopic treatment [5, 6, 8].

The purpose of this paper is to report operating methods, findings, and overall results for separated ossicles of the lateral malleolus that were treated by arthroscopic excision.

## Materials and methods

**Fig. 1:**
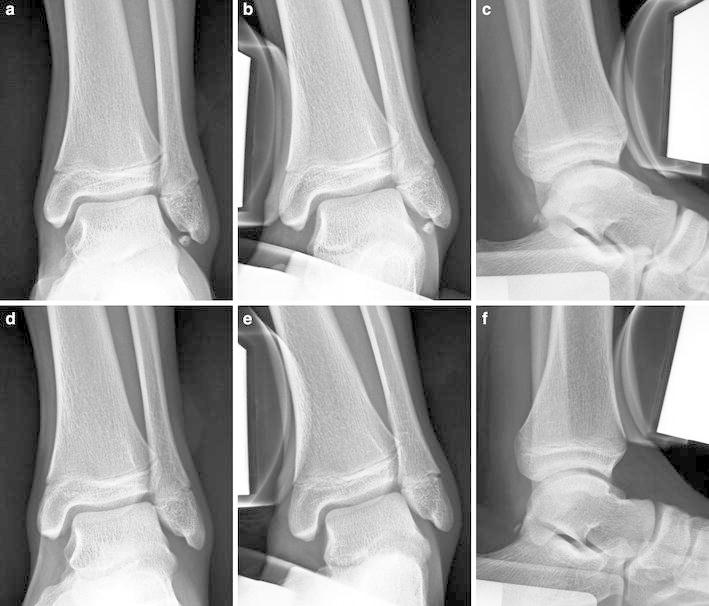
Preoperative radiographs (**a**–**c**) and postoperative radiographs (**d**–**f**) (Case 19). Anteroposterior radiograph (**a**) showing a small ossicle (5 mm in diameter). Varus stress radiograph (**b**) and anterior stress radiograph (**c**) showing no instability of the ankle. Anteroposterior radiograph (**d**) showing a ossicle excised. Varus stress radiograph (**e**) and anterior stress radiograph (**f**) showing no increase of ankle instability

Indications for arthroscopic excision of separated ossicles of the lateral malleolus complied with the conditions below, on the basis of clinical symptoms, plain radiographs, and stress radiographs (Fig. 1).

1. The main complaint is pain alone, with no, or only slight, sensations of ankle instability.
2. On stress radiographs, there is no, or relatively little, ankle instability (talar tilt angle ≤10°, and anterior displacement <10 mm).
3. The size of ossicle is small on the anteroposterior plain radiograph (longitudinal diameter ≤5 mm), and the possibility of increasing ankle instability postoperatively seems to be minor.

If, on the other hand, ankle instability was apparent from clinical or imaging findings, and if it seemed possible that postoperative ligament dysfunction would result because of the large size (>5 mm) of ossicles, other open treatment was chosen [2, 3, 5, 7].

Between 1990 and 2009, operative treatment was performed on 111 ankles of 107 patients for separated ossicles of the lateral malleolus. Excision of the ossicle (and repair or reconstruction of the lateral ligament of the ankle) was conducted on 75 ankles of 72 patients, arthroscopic excision of the ossicle was performed on 20 ankles of 19 patients, and fusion of the ossicle was performed on 16 ankles of 16 patients. Arthroscopic treatment was performed on 12 ankles of 12 males and on eight ankles of seven females. The average age at the time of operation was 17.6 (range 8–35) years. Mean postoperative followup was 50.2 (range 29–126) months. This study was approved by our Institutional Review Board.

Chief complaints consisted of pain at the tip of the lateral malleolus during exercise in all cases; slight sensations of ankle instability were noted in three cases. All patients acknowledged local tenderness surrounding the site of the ossicles. A clear history of trauma was noted for 12 ankles (sprains of 11 ankles, a traffic accident for one ankle). Pain in seven ankles was concluded to have been caused by overuse, and the cause was unclear for one ankle. All patients received nonoperative treatment for more than three months. However, because symptoms did not improve, arthroscopic treatment was performed.

Stress radiographs were taken by use of a TELOS device (TELOS, Weiterstadt, Germany). The talar tilt angle and anterior displacement were measured before surgery and at last followup examination. The talar tilt angle was defined as the angle between the articular surfaces of the tibia and talus on the anteroposterior varus stress radiograph. Anterior displacement was determined as the shortest distance between the posterior lip of the tibia and the talar dome on the lateral anterior stress radiograph [9].

The results from surgery were based on the Japanese Society for Surgery of the Foot (JSSF) ankle/hindfoot scale [10]. For all patients, pre and postoperative JSSF ankle/hindfoot scales were assessed retrospectively from the medical records (Table 1).

**Table 1:** Patient data

Case	Gender	Age	Pain	Sensation of instability	Cause of injury	Preoperative TTA (degrees)	Postoperative TTA (degrees)	Preoperative AD (mm)	Postoperative AD (mm)	Preoperative JSSF	Postoperative JSSF	Sports	Time to recover (weeks)
1	F	14	(+)	(−)	Sprain	10	9	7	8	80	100	Soccer	6
2	M	13	(+)	(−)	Sprain	9	9	8	7.5	80	100	Soccer	6
3	F	16	(+)	(±)	Sprain	5.5	7	9	12	70	92	Gymnastics	8
4	M	25	(+)	(−)	Traffic accident	3	5	5	5	75	85	(-)	8
5	F	12	(+)	(−)	Overuse	2	3	4.5	5	77	100	Basketball	6
6	F	8	(+)	(−)	Overuse	9	7	6	6	77	100	Basketball	6
7	M	19	(+)	(±)	Sprain	10	7	3	4	77	90	Ski	8
8	F	15	(+)	(−)	Sprain	5	6	7	6	80	100	Tennis	7
		21	(+)	(−)	Sprain	5	5	6.5	6	80	100	Tennis	8
9	F	20	(+)	(−)	Sprain	7	6	6	7	78	88	Athletics	8
10	M	17	(+)	(−)	Overuse	5	5	6.5	6	78	100	Soccer	8
11	M	16	(+)	(−)	Sprain	5	5	6	5	80	100	Baseball	8
12	M	24	(+)	(−)	Overuse	6	6	5	7	75	100	Soccer	8
13	F	15	(+)	(−)	Sprain	4	3	2	2	80	100	Ballet	6
14	M	15	(+)	(−)	Overuse	4.5	6	7	7	77	100	Baseball	8
15	M	16	(+)	(±)	Sprain	10	10	8	7	78	100	Basketball	8
16	M	18	(+)	(−)	Unclear	5	6	5.5	6	75	100	Baseball	8
17	M	35	(+)	(−)	Overuse	4	5	4	4	75	88	(-)	8
18	M	17	(+)	(−)	Overuse	7	6.5	6	7	80	100	Soccer	8
19	M	15	(+)	(−)	Sprain	5	4	6	4.5	80	100	Baseball	6

*TTA* talar tilt angle, *AD* anterior displacement, *JSSF* Japanese Society for Surgery of the Foot ankle/hindfoot scale

For talar tilt angle, anterior displacement, and JSSF scale, paired *t* tests were used for statistical analysis; results were regarded as statistically significant for values of *p* < 0.05.

### Operative technique

The patient was placed in a supine position under general anesthesia with a pneumatic tourniquet on the thigh. During the operation, intracapsular working space was maintained by using a joint-irrigation system with approximately 60 mmHg pressure. No distraction devices were applied to the ankle. A 2.7-mm 30° arthroscope was inserted into the ankle joint via the anterolateral portal. The accessory anterolateral portal was established approximately 10 to 15 mm distal from the anterolateral portal, in the superior margin of the anterior talofibular ligament, and instruments were inserted using this portal [11].

**Fig. 2:**
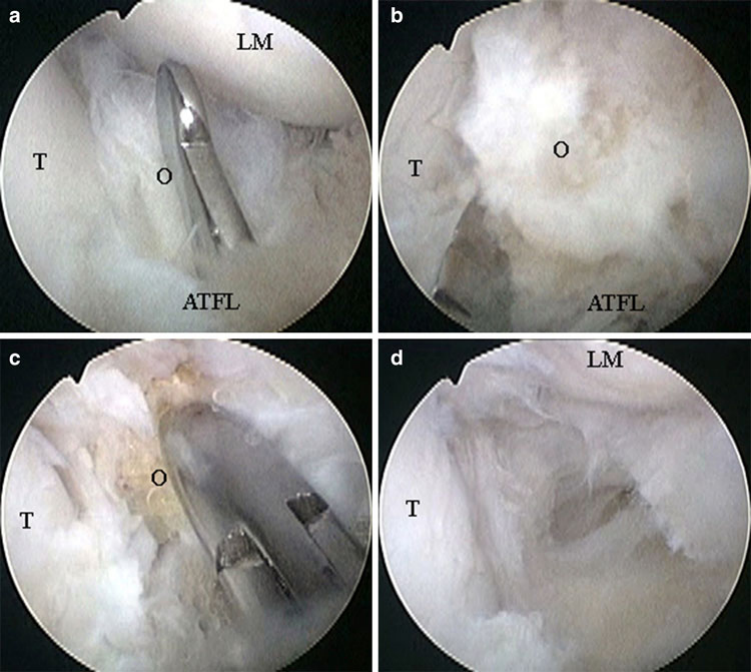
Arthroscopic findings (Case 19). **a** Palpation using the probe. **b** Dissecting the ossicle from the surrounding ligament fibers by use of a banana knife. **c** Removing the ossicle piece by piece with a grasper. **d** After excision of the ossicle. (*O* ossicle, *T* talus, *ATFL* anterior talofibular ligament *LM* lateral malleolus)

Because the ossicle was partially or completely embedded within the fibers of the anterior talofibular ligament, its size and location were confirmed by palpation with the probe (Fig. 2a). A 3.5-mm full-radius shaver was inserted, and resection of the surrounding inflamed synovial tissue was performed to obtain visual space. The ossicle was then carefully dissected from the surrounding ligament fibers by use of a banana knife, but the procedure was maintained as noninvasive as possible by not disrupting the continuity of ligament fibers (Fig. 2b). The ossicle was removed piece by piece with a grasper (Fig. 2c). Surgery was complete when no remains of the ossicle could be confirmed by palpation with the probe or by use of an intraoperative radiograph (Fig. 2d). The ossicles were carefully and marginally dissected from the surrounding ligament fibers, and we were able to minimize damage to the ligaments for all patients.

Postoperatively, the ankle was immobilized in a walking cast for three weeks. When the cast was removed an ankle brace was applied, and patients were permitted to resume daily activities on a gradual basis. The brace was kept on postoperatively until six weeks had passed. After a period of approximately six to eight weeks postoperatively, patients were allowed to return to sports activities.

## Results

All patients recovered sufficiently to participate at their original levels of activity within eight weeks of surgery. Seventeen patients (18 ankles) were athletes; for these average time to return to sports was 7.3 weeks (range 6 to 8). There were no other complications, for example postoperative skin trouble, infections, or nerve injuries. Of 20 ankles, no pain occurred in sixteen and slight pain was noticed in four during exercise. For three ankles sensations of ankle instability were experienced, for two ankles there were no symptoms, and for one ankle (Case 3) symptoms were increased.

With regard to arthroscopic findings, it was confirmed for all patients that the ossicles were partially or completely embedded within the fibers of the anterior talofibular ligament. Slight hypertrophy of the white synovial tissue surrounding the ossicles was confirmed, but there were no clear findings of impingement.

The mean talar tilt angle changed from 6.1° ± 2.4° preoperatively to 6.0° ± 1.8° postoperatively (*p* = 0.93), and the mean anterior displacement changed from 5.9 ± 1.7 mm preoperatively to 6.1 ± 2.0 mm postoperatively (*p* = 0.42), indicative of minimum alteration between the pre and postoperative values. The average JSSF ankle/hindfoot scale improved from 77.6 ± 2.6 points preoperatively to 97.2 ± 5.2 points postoperatively (*p* < 0.01) (Table 1).

## Discussion

Because of the spread of, and progress in, ankle arthroscopy in recent years, several reports have shown that arthroscopic excision of ossicles of the malleoli of the ankle has achieved good results [5, 6, 8]. It is generally accepted that arthroscopic treatment is less invasive than open operative treatment and it is possible to achieve relatively early resumption of daily and sports activity. In this discussion we consider the clinical results of arthroscopic excision of separated ossicles of the lateral malleolus.

**Fig. 3:**
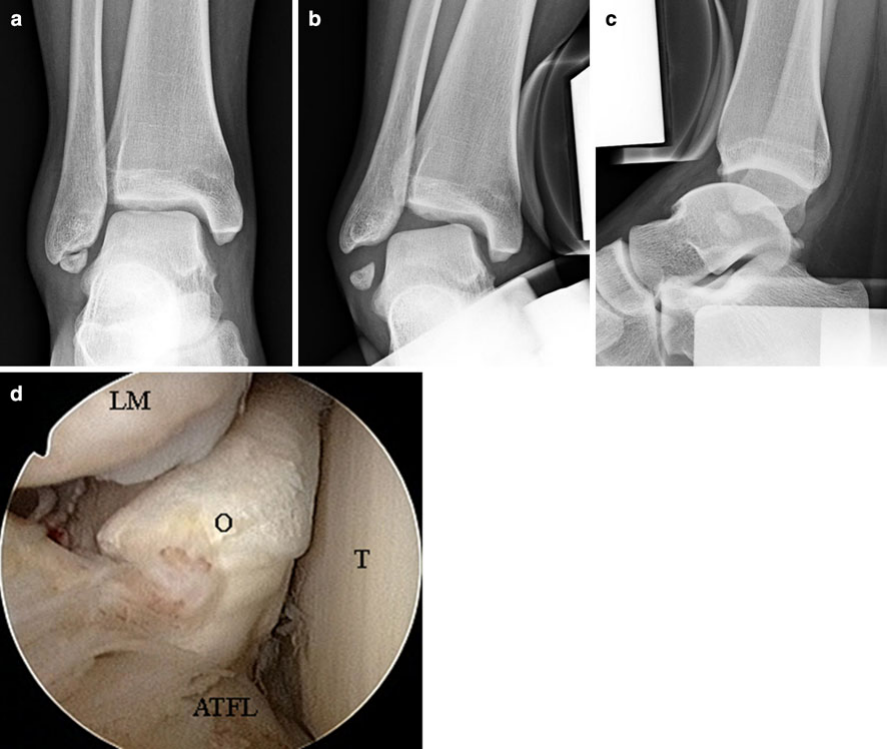
Case showing no indication for arthroscopic excision (15-year-old female). Anteroposterior radiograph (**a**) showing a large ossicle. Varus stress radiograph (**b**) and anterior stress radiograph (**c**) showing high instability of the ankle and an opening between the ossicle and lateral malleolus. Arthroscopic findings (**d**) showing a large gap between the ossicle and lateral malleolus (*O* ossicle, *T* talus, *ATFL* anterior talofibular ligament, *LM* lateral malleolus)

With regard to location of the separated ossicle of the lateral malleolus, Hasegawa et al. reported that the ossicles were embedded partially or completely within the fibers of the anterior talofibular ligaments and that, in arthroscopic findings, most ossicles were connected to the short fibers of the posterior talofibular ligament [5]. Han et al. reported arthroscopic treatment of 24 ossicles of the malleoli of the ankle, and no cases of instability of the ankle if debridement was performed carefully to minimize damage to the ligament [6]. In this procedure, however, no matter how carefully the ossicles may have been removed, cutting of the ligament fibers could not be avoided. The anterior talofibular ligament connected to the ossicle is extremely important for maintaining the stability of the ankle joint. When ossicles were large and ankle instability was found, as in the case shown in Fig. 3, the procedure could not be used. In these cases, open treatment for excision of the ossicle and reconstruction of the lateral ligament of the ankle were performed to reliably ensure proper ligament function.

In our series, the postoperative JSSF scale was 97.2 points. All patients recovered sufficiently and were able to participate at their original levels of activity within eight weeks, which was sooner than for reports of open treatment [3, 7]. With regard to postoperative care, Han et al. reported no cases confirming instability of the ankle in the absence of postoperative immobilization [6]. However, with regard to cutting of ligament fibers in this procedure, as mentioned above, we considered that postoperative treatment should be the accepted method conforming to treatment of acute ankle sprain, to prevent the postoperative occurrence of any ligament dysfunction. With regard to the strong demands of one patient (Case 3, a female gymnast), cast immobilization and an ankle brace were not applied and she was allowed to return to her sports activity as early as possible. However, although her pain disappeared, ankle instability increased postoperatively. As a result, during postoperative treatment cast immobilization was performed for three weeks, and an ankle brace was then applied for another three weeks [12, 13]. However, several recent reports have shown that good results can be obtained even if the period of cast immobilization is short (seven to 10 days) or absent, as long as an ankle brace is used in the early period [14, 15]. Therefore, in the future, we will determine whether it is safe to modify the postoperative treatment so that fixation with an ankle brace is the major treatment, thus minimizing the period of cast immobilization.

With regard to the size of the separated ossicles of the lateral malleolus, since 1990 we have measured the size of the ossicle on anteroposterior plain radiographs to help determine the best operative approach. Because of progress in diagnostic imaging by CT and MRI in recent years, however, it has become possible to obtain more detailed descriptions, and to determine the size and location of ossicles. As a result, several reports have been published describing evaluation of the ossicles by use of these imaging techniques [6, 16, 17]. Therefore, we consider that CT and MRI should be adopted to update the accuracy of our diagnostic imaging in the future.

In conclusion, arthroscopic excision of separated ossicles of the lateral malleolus achieved good results and was performed with a minimum of incisions. It was possible to allow relatively early resumption of daily and sports activity, as mentioned above. However, we believe postoperative treatment should conform to the accepted method of treatment of an acute ankle sprain, for example fixation with an ankle brace, to minimize the occurrence of ligament dysfunction.

## References

[1] Powell HDW (1961). Extra center of ossification for the medial malleolus in children: incidence and significance. J Bone Joint Surg Br.

[2] Griffiths JD, Menelaus MB (1987). Symptomatic ossicles of the lateral malleolus in children. J Bone Joint Surg Br.

[3] Berg EE (1991). The symptomatic os subfibulare. Avulsion fracture of the fibula associated with recurrent instability of the ankle. J Bone Joint Surg Am.

[4] Ogden JA, Lee J (1990). Accessory ossification patterns and injuries of the malleoli. J Pediatr Orthop.

[5] Hasegawa A, Kimura M, Tomizawa S, Shirakura K (1996). Separated ossicles of the lateral malleolus. Clin Orthop Relat Res.

[6] Han SH, Choi WJ, Kim S, Kim SJ, Lee JW (2008). Ossicles associated with chronic pain around the malleoli of the ankle. J Bone Joint Surg Br.

[7] Kono T, Ochi M, Takao M, Naito K, Uchio Y, Oae K (2002). Symptomatic os subfibulare caused accessory ossification: a case report. Clin Orthop Relat Res.

[8] Bonnin M, Bouysset M (1999). Arthroscopy of the ankle: analysis of results and indications on a series of 75 cases. Foot Ankle Int.

[9] Karlsson J, Bergsten T, Lansinger O, Perterson L (1988). Reconstruction of the lateral ligaments of the ankle for chronic lateral instability. J Bone Joint Surg Am.

[10] Niki H, Aoki H, Inokuchi S, Ozeki S, Kinoshita M, Kura H, Tanaka Y, Noguchi M, Nomura S, Hatori M, Tatsunami S (2005). Development and reliability of a standard rating system for outcome measurement of foot and ankle disorders I: development of standard rating system. J Orthop Sci.

[11] Ferkel RD (1996). Arthroscopic surgery: the foot and ankle.

[12] Pijnenburg AC, Van Dijk CN, Bossuyt PM, Marti RK (2000). Treatment of ruptures of the lateral ankle ligaments: a meta-analysis. J Bone Joint Surg Am.

[13] Yamamoto H, Ishibashi T, Muneta T, Furuya K (1993). Nonsurgical treatment of lateral ligament injury of the ankle joint. Foot Ankle.

[14] Beynnon BD, Renström PA, Haugh L, Uh BS, Baker H (2006). A prospective, randomized clinical investigation of first-time ankle sprains. Am J Sports Med.

[15] Jones MH, Amendola AS (2007). Acute treatment of inversion ankle sprains: immobilization versus functional treatment. Clin Orthop Relat Res.

[16] Nakasa T, Fukuhara K, Adachi N, Ochi M (2006). Evaluation of anterior talofibular ligament lesion using 3-dimensional computed tomography. J Comput Assist Tomogr.

[17] Kim BS, Choi WJ, Kim YS, Lee JW (2010). The effect of an ossicle of the lateral malleolus on ligament reconstruction of chronic lateral ankle instability. Foot Ankle Int.

